# Local Adaptation of Aboveground Herbivores towards Plant Phenotypes Induced by Soil Biota

**DOI:** 10.1371/journal.pone.0011174

**Published:** 2010-06-17

**Authors:** Dries Bonte, Annelies De Roissart, Martijn L. Vandegehuchte, Daniel J. Ballhorn, Thomas Van Leeuwen, Eduardo de la Peña

**Affiliations:** 1 Department of Biology, Ghent University, Ghent, Belgium; 2 Department of Plant Biology, University of Minnesota, St. Paul, Minnesota, United States of America; 3 Department of Crop Protection, Ghent University, Ghent, Belgium; Field Museum of Natural History, United States of America

## Abstract

**Background:**

Soil biota may trigger strong physiological responses in plants and consequently induce distinct phenotypes. Plant phenotype, in turn, has a strong impact on herbivore performance. Here, we tested the hypothesis that aboveground herbivores are able to adapt to plant phenotypes induced by soil biota.

**Methodology and Principal Findings:**

We bred spider mites for 15 generations on snap beans with three different belowground biotic interactions: (i) no biota (to serve as control), (ii) arbuscular mycorrhizal fungi and (ii) root-feeding nematodes. Subsequently, we conducted a reciprocal selection experiment using these spider mites, which had been kept on the differently treated plants. Belowground treatments induced changes in plant biomass, nutrient composition and water content. No direct chemical defence through cyanogenesis was detected in any of the plant groups. Growth rates of spider mites were higher on the ecotypes on which they were bred for 15 generations, although the statistical significance disappeared for mites from the nematode treatment when corrected for all multiple comparisons.

**Conclusion/Significance:**

These results demonstrate that belowground biota may indeed impose selection on the aboveground insect herbivores mediated by the host plant. The observed adaptation was driven by variable quantitative changes of the different separately studied life history traits (i.e. fecundity, longevity, sex-ratio, time to maturity).

## Introduction

Host plant specialisation in herbivorous species is the rule rather than the exception [1]. This tight association is the result of historical arms races between plants and their antagonists, and the mechanism behind the extraordinary diversity of plant-associated insects. The high level of host plant specialisation is the result of the enormous variation in plant structure and biochemistry [2] and driven by associated variation in insect herbivore performance among plant species [3]. In addition, spatial variation in both biotic and abiotic conditions generates strong variation in plant phenotypes, either by phenotypic plasticity or by natural selection (geographic mosaics of selection; [4]). Within-species genotypic variation is therefore also likely to induce selection on herbivore performance. While there is compiling evidence of genotype-associated changes in herbivore performance and abundance [5], , only few studies have documented local adaptation of herbivores to either single plant genotypes within a species or to individual plants phenotypes [14], [15], [16], [17].

Studies investigating herbivore local adaptation often neglect plant belowground interactions. However, over the last decade abundant empirical evidence has been gathered, indicating that plants intimately integrate above- and belowground parts of ecosystems and therefore, interactions occurring at one side of the soil surface cannot be understood without taking into account what occurs at the other [17], [18], [19]. Mutualistic endophytic fungi (e.g., arbuscular mycorrhizal fungi) and root-feeders (e.g., root-feeding nematodes) are two of the soil functional groups that interact directly with plants roots. They are able to cause strong changes in plant biomass and nutrient allocation, water content and the concentration of chemical defence compounds [17], [18], [20], [21], [22], [23]. These changes in plant quality do not operate in a mutually exclusive way and may strongly interact with behaviour and population dynamics of associated arthropod herbivores and mutualists [24], [25], [26]. For instance, the performance of specialist herbivores which can cope with chemical defence traits is predominantly affected by water stress and mechanical defence while in other species, multiple defence mechanisms determine feeding and performance [27], [28], [29].

Although mycorrhizal fungi are generally considered plant mutualists, it has been demonstrated that, depending on both plant and fungus species identity and plant age, mycorrhizae can also negatively affect plant performance [30], [31], [32], [33]. In a meta-analysis of 34 studies, [34] found a marginally significant overall positive effect of mycorrhizal fungi on insect herbivores. However, there were large differences between feeding guilds. Based on 4 studies, they conclude that the performance of mesophyll feeders, such as spider mites, is lower on mycorrhizal plants. Recently however, [35] demonstrated that AMF benefit plant growth and as such increased spider mite performance.

While root-feeding nematodes are notorious for their devastating effects on crop plants [36], [37], it has been shown that low amounts of root grazing by nematodes can indirectly enhance plant performance [38], [39]. Effects of root-feeding nematodes on aboveground herbivores can be negative due to the induction of systemic defences [28], or by the lowering of amino-acid contents of leaves [40]. Positive effects can for instance arise as a result of the destruction by root-feeding nematodes of production sites of secondary metabolites in the roots [41].

The selective forces that soil biota exert through changes in plant characteristics (either through changes in plant phenotype, plant genotype or plant species composition) on these aboveground herbivores are therefore a fundamental factor to understand the functioning of terrestrial communities. Paradoxically, the importance of above-belowground interactions is well acknowledged from a community ecology perspective but the consequences for evolutionary dynamics, although suggested, have hardly been addressed [42], [43].

The spider mite *Tetranychus urticae* (Acarina: Tetranichidae) is a cosmopolitan aboveground herbivore and a devastating pest species on a wide variety of naturally occurring plant species as well as crops [44], [45]. The species is characterized by a haplodiploid life cycle and known for its extreme potential for local adaptation towards different plant species [46], [47]. Here, we determined in first instance how arbuscular mycorrhizal fungi (AMF) and belowground root-feeding nematodes (*Pratylenchus penetrans*; Tylenchida: Pratylenchidae) induce changes in the performance of a plant species (Common bean or snap bean; *Phaseolus vulgaris*). We specifically emphasised on traits that may induce negative effects on growth of aboveground herbivores through the action of soil biota (reviewed in [19], [21], [43], [48]). Besides classically reported changes in nutrient and water content, we also determined whether chemical defences are induced in relation to the belowground treatment. Both lima bean (*Phaseolus lunatus*) and snap bean (*P. vulgaris*) have been reported to produce release of toxic hydrogen cyanide from preformed cyanide-containing compounds (cyanogenesis; [49], [50], [51]) after attack of aboveground, but presumably also belowground antagonists.

Secondly, we established selection lines of spider mites for 15 generations on plants that had been exposed to three different treatments: (i) sterile soil to serve as control, (ii) soil inoculated with AMF, and (iii) soils containing root-feeding nematodes. In reciprocal breeding experiments we investigated whether local adaptation occurs of herbivores to plant phenotypes induced by different belowground biotic agents. We subsequently use the artificial and non-coevolved bean-mite-nematode/AMF system as a test case to investigate whether belowground biota are able to induce adaptive, evolutionary responses in their aboveground counterparts.

According to literature, we hypothesise that the performance of *P. vulgaris* plants will be moderately lowered or even increased in the presence of AMF, while it is lowered by root-feeding nematodes. We furthermore predict that *T. urticae* fitness will be higher on the type of plants they were bred on for 15 generations, compared to plants that differ in their belowground treatment.

## Materials and Methods

### The model system

We used a genetically diverse source population of *Tetranychus urticae* [52], which has been kept in culture on common bean (*Phaseolus vulgaris* L. cultivar Prelude) for nearly ten years. We retained the host plant for our experiment, but introduced variation in soil biotic composition to create different bean phenotypes induced by different soil organisms (nematodes vs. AMF). The experiment compared the performance of three lines of spider mites reared on stock plants prepared as follows. For the reference line, bean plants were grown on standard sterilized (120°C, 120 minutes, 1 Atm) potting soil in five 5 liter trays of 15×15×35 cm (15 plants/tray). This soil treatment is further referred to as ‘control’. To introduce AMF, we inoculated sterilized soil with a commercial mixture of arbuscular mycorrhizal fungi (MycoGrow™) of *Glomus mossae* (five identical trays as for controls). We followed a modification of the procedure advised by the manufacturer and inoculated plants by watering plant-trays with 500 ml of demineralised water containing 1 g of the mycorrhizal inoculum. To infect beans with nematodes, we added 5000 *Pratylenchus penetrans* (Tylenchida: Pratylenchidae) to five trays filled with sterilized soil. Trays containing bean plants were watered twice a week with tap water. Every three weeks, we used one-month old plants from these treatments for the inoculation with spider mites and refreshed the stock population immediately with new seeds that were allowed to grow for another month and to be colonized by the soil biota before the inoculation with spider mites (see below).

### The reciprocal breeding experiment

We first allowed AMF and nematode populations to establish on plants for one month. Of each treatment group, randomly selected stock beans from the five trays were carefully transplanted (keeping root damage to a minimum) to the growth chambers for inoculation with mites. We repeated this transplantation every three weeks allowing each time for similar degrees of nematode and AMF infection. Thus, plants were refreshed in the growth chambers every third week, just before complete wilting.

The *T. urticae* source population was split into three selection lines (control, AMF, and nematode). Instead of keeping small populations on single leaves (sensu [46], [47]), we chose to retain large selection line populations (N≫10000) on 10–15 simultaneously grown bean plants with an identical belowground treatment for 15 generations (September 2008 -April 2009). The rationale behind this setup was (i) to preclude changes in leaf quality due to induced damage by leaf harvestings and (ii) to guarantee sufficient genetic variation within each of the three selection lines and to avoid genetic drift. During the experiment, mites were kept under controlled ambient conditions (28°C, 60%RH and 16∶8 day:night light regime).

At the end of the induced selection, a reciprocal breeding experiment of females from the three selection lines on plants from the different belowground treatments was established. From each selection line ten inseminated females were selected from different plant leaves. For logistical reasons and since we only used one mite strain (see above), we considered these individual females from the three subpopulations on plants with a different belowground treatment to be independent replicas. Both highly genetically diverse starting populations and the multiple bean plants used during selection (which can be expected to experience various levels of belowground interactions; as such averaging stochastic changes in plant quality) render this setup valid.

Offspring from the selected females was raised for two generations on leaf discs (1 cm^2^) on control plants (i.e., those grown on sterile soil) in order to correct for possible maternal effects [47]. Leaf discs were placed with the abaxial part upwards on moistened filter paper to prevent mites from escaping and to maintain leaf turgor. The mite lines were subsequently highly inbred because we allowed only sib-mating (estimated inbreeding coefficient: 0.9; [46]). Four individual females per F2 generation were tested for performance during and after juvenile development. Performance was tested on leaves from the three different plant treatment groups in a climate chamber with conditions adjusted as described above. Males from the same kin group were added in the deutonymph life stage to guarantee for sexual reproduction. For each initially selected female from the three selection lines, we consequently assessed reaction norms of 12 genetically highly similar F2-offspring for their performance on bean of the three different belowground treatments. The following life history parameters were recorded daily: mortality, developmental stage (i.e. developmental time from first nymphal stage till maturity), fecundity (number of eggs) and gender of the offspring (n = 2017). Because spider mites deposit the majority of their eggs during the first ten days after maturity [35], we monitored fecundity only during that period. Mites that died due to drowning were excluded from the analyses.

Single life history parameters may not fully allow the detection of local adaptation [53]. Therefore, we additionally simulated an integrated fitness measure, the rate of intrinsic growth (*r_m_*). This was estimated from the life history parameters according to the formula: 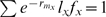 with *l_x_* survival till maturity *x*, *f_x_* the number of female offspring at age *x*. Because we found no mortality of mature females during the considered oviposition time window, we adopted a slightly modified measure of growth rate by not taking into account total longevity. The measure consequently represents the contribution of each female to the number of females in the subsequent generation.

### Belowground biota colonization and plant performance

Levels of infection by AMF and root nematodes were evaluated in 25–30 one-month old plants bred for the selection experiment (so, plants of the same age as used in the reciprocal breeding experiment). We evaluated plant traits and the biota colonisation at the end of the selection experiment. Bean plants were uprooted and washed until all rests of substrate were removed. Roots were cut in 1 cm fragments and subsequently, nematodes were extracted using the Baermann funnel technique [54]. Root fragments not used for nematode extraction were stained following the technique of [55] and assessed for AMF colonization according to the grid-intersect method described by [56] using a microscope. Nematode colonization was only found in the nematode treatment with of 1.31±0.75; (mean ± SE) nematodes·g^−1^ of soil and 44.3±12.2 nematodes·g^−1^ of root (n = 27). Similarly, only bean plants from the AMF treatment were colonized by AMF (with an average percentage of root colonization of 21.4±12.3 (n = 28).

In order to asses plant growth related parameters, at the end of the experiment, we harvested ten bean plants from each soil treatment. Above- and belowground biomass were measured in first instance by weighing fresh weight and dry weight (40 hours drying in an air-flow oven at 70°C). Water content of shoots and roots was calculated from the relative difference between fresh and dry weight. For another three bean plants, we analysed nitrogen content by ISO 5983-2 [57]. Phosphorous-content was analysed by colorimetry (EC L279/15 20.12.71). Potential changes in chemical defences were assessed by quantification of the cyanogenic potential (HCNp) [51]. Cyanogenesis, that is, the wound- induced release of toxic hydrogen cyanide from preformed cyanide-containing compounds is one of the best analyzed direct defenses of beans belonging to the genus *Phaseolus*. Both lima bean (*Phaseolus lunatus*) and snap bean (*P. vulgaris*) have been reported to produce this type of defence compounds [49], [50], [51]. For another ten plants per treatment, we therefore selected defined leaf developmental stages to reduce variability of leaf texture and HCNp due to ontogeny. We selected unfolded leaves three positions down the apex. One leaf per plant individual was used for analyses (see [51] for a detailed description of HCNp quantification).

#### Statistical analysis

Plant performance parameters were analysed using analysis of variance with soil treatment as the independent factor. Full factorial linear models were used to infer differences in the mean life history traits according to their original selection line (three levels) and treatment (three levels). We controlled for similarity due to common origin by including maternal F2 genotype and its interaction with the treatment as random effects. Time after maturity was included as repeated measurement random effect (compound symmetry correlation structure) in the models to analyze effects on daily fecundity. Survival till maturity and offspring sex ratio were analysed by generalized mixed models with binomial error structure and a logit-link, controlled for potential overdispersion by modelling residuals as R-side random effects. Daily fecundity followed a Poisson distribution and was similarly modelled by using a log-link function. Satterthwaite procedure was applied to approximate the effective degrees of freedom. Analyses were conducted with SAS 9.1 (SAS Institute Inc 2006) by using the GLIMMIX procedure. Bootstrapped *r_m_*-values were analyzed by generating 99% confidence intervals on the simulated average values and by performing two-way Anova on the simulated data. We performed posthoc Tukey tests to correct pair wise differences between treatments within each of the three selection lines.

## Results

### Plant performance

**Figure 1:**
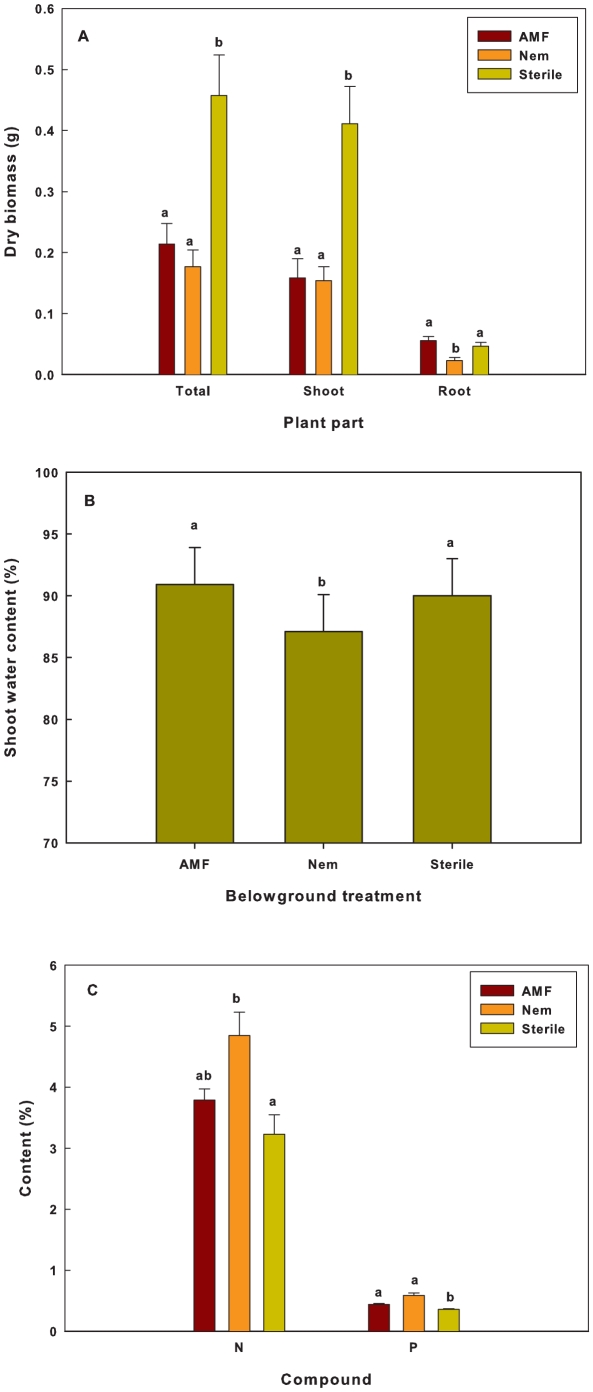
Effects of the soil treatment on plant performance. A): plant dry biomass; B: shoot water content; C: N and P-content. Equal notations indicate non-significant contrast for the respective plant performance measurements. Values marked with the same letter symbol do not differ significantly (P>0.05) after Tukey correction.

The belowground treatment of plants had a significant effect on plant biomass and nutritional composition (Table 1). Both belowground treatments, AMF and nematodes, had a detrimental effect on total and aboveground plant biomass (Fig. 1A). Plants with nematodes were characterised by a lower belowground biomass compared to AMF and control plants (Fig. 1A). Conversely, biomass allocation to roots (i.e. the ratio below/aboveground biomass) was highest in mycorrhizal plants (0.29±0.03se) compared to plants from the nematode treatment (0.13±0.02se) and the sterile-soil (0.12±0.03se) treatment. Water content only differed among the treatments for shoots (Table 1). While water content for roots averaged 86.23%±0.6%se, shoot water content was on average 3% lower in beans treated with nematodes (Fig. 1B). The belowground treatment resulted in differences in nitrogen and phosphor content (Table 1), with highest N-concentration in plants treated with nematodes and lowest P-levels in controls (Fig. 1C). No detectable levels of cyanogenic precursors were found in any of the plants.

**Table 1:** Results of ANOVA of the measured plant biomass and plant quality variables in relation to the soil treatment.

Plant performance measure	Num d.f., Den. d.f.	*F*	*P*
Total biomass (g)	2,27	12.44	<0.0001
Shoot biomass (g)	2,27	13.69	<0.0001
Root biomass (g)	2,27	8.86	<0.0012
Ratio root/total biomass	2,27	14.49	<0.0001
Root water content (%)	2,27	2.18	0.132
Shoot water content (%)	2,27	42.95	<0.0001
N-content (% dry weight)	2,6	7.09	0.026
P-content (% dry weight)	2,6	17.81	0.003

### Mite performance after selection

After selection, mites differed significantly in the measured life history parameters according to the selection line, their soil treatment and the interaction between both (Table 2). When combined to one integrative measure (the simulated growth rate), performance was higher on treatments that matched the belowground selection treatment, indicating local adaptation of mites to the belowground biota. Here-under, we provide details for the separate parameters and the resultant growth rate.

**Table 2:** Results for fixed effects from mixed linear models with time to maturity, female survival rates till maturity, daily fecundity, sex ratio and simulated growth rate as response variable*.

Factor	Num df	Den df	*F*	*P*
**Time to maturity**				
Selection line	2	364	48.34	<0.001
Sex	1	35	66.34	<0.001
Soil treatment	2	18	2.98	0.077
Selection line x Sex	2	364	0.81	0.812
Selection line x Soil treatment	4	364	15.25	<0.001
Sex x Soil treatment	2	18	0.19	0.882
Selection line x Soil treatment x Sex	4	364	2.24	0.064
**Female survival**				
Selection line	2	61	3.10	0.052
Soil treatment	2	17.64	0.66	0.532
Selection line x Soil treatment	4	61	19.79	<0.001
**Daily fecundity**				
Selection line	2	37.2	2.28	0.116
Soil treatment	2	39.7	5.10	0.011
Selection line x Soil treatment	4	37.1	2.67	0.047
**Sex Ratio**				
Selection line	2	90	1.73	0.183
Soil treatment	2	90	2.10	0.128
Selection line x Soil treatment	4	90	6.05	<0.001
**Growth rate**				
Selection line	2	415	88.95	<0.001
Soil treatment	2	415	385.16	<0.001
Selection line x Soil treatment	4	415	969.2	<0.001

*Gaussian error distributions were modelled for time to maturity, Poisson errors for daily fecundity, binomial errors for female survival and sex ratio.

#### Time to maturity

**Figure 2:**
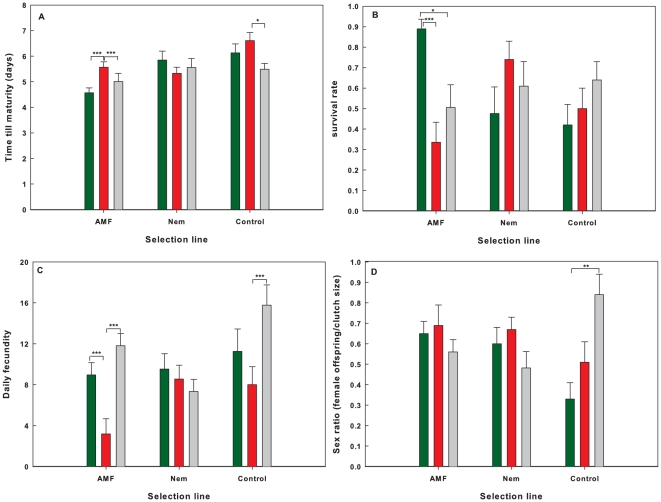
Effects of soil treatment on the selected life history parameters (mean values ± SE) for mites of the three selection lines. Green bars: mites developed on AMF-treated plants; Red bars: mites developed on Nematode-treated plants; Grey bars: mites developed on Control plants (no belowground biota). A: time to maturity (females), B: survival rate of females, C: daily fecundity, D: sex ratio (females/total clutch size). Statistical significant differences within selection lines after Tukey-corrections: *: P<0.05; **: P<0.01; ***: P<0.001.

Males developed under the prevailing lab conditions in on average 5.61±0.11se days till the adult life phase. This is on average 0.46 days faster than females under the same conditions (table 2). A significant selection line x soil treatment interaction was observed, with shortest developmental times for mites with matching selection line-treatment combinations (Fig. 2A). Significant pairwise differences were recorded between the AMF and both nematode and control treatment in the AMF selection line and between the nematode and control treatment in the control selection line (Fig. 2A). In the AMF selection line, developmental time was also higher in mites reared on beans on a sterile soil, compared to those treated with nematodes.

#### Survival

The interaction between selection line and soil treatment was highly significant for survival rate (table 2). In general, survival was highest when the soil treatment matched the selection line, but differences within selection lines were only significant in the AMF-line after correction for multiple comparisons (Fig. 2B).

#### Fecundity

Daily fecundity differed among mites from the different selection lines, the soil treatment and their interaction (table 2). The average daily fecundity was highest on the control selection line compared to the other lines (*t*>3.95; *P*<0.001) and higher on the nematode line compared to the AMF line (*t* = 2.00; *P* = 0.45). According to the soil treatment, daily fecundity increased from nematodes (6.17±1.01), over AMF (8.93±1.01) to controls (9.50±0.97). The latter two differed significantly from the nematode treatment (*t*>2.31; *P*<0.05). The interaction between selection line and the soil treatment (Fig. 2C) was especially prominent for mites from the AMF selection line, with significantly lower fecundity on the nematode treatment (*t*>5.77; *P*<0.001) and for those originating from the control line with significantly higher fecundity on the control treatment compared to the nematode treatment (*t* = 3.13; *P*<0.001).

### Sex ratio

The average proportion of females within clutches was 0.59±0.12. No overall differences among soil treatments or selection lines were recorded. The proportion of females within clutches showed a significant selection line x treatment interaction (table 2). Pronounced differences were only observed for the control selection line (Fig. 2D) with significantly higher proportions of females in the control treatment (0.84±0.10) compared to the AMF treatment (0.33±0.08).

### Growth rate

**Figure 3:**
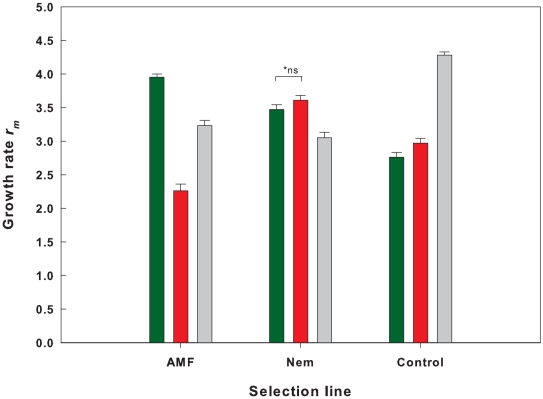
Effects of the soil treatment on measure growth rate r_m_. The mean integrated fitness measure growth rate r_m_ relates to the number of female offspring by one female per generation for mites from each of the three selection lines. Green bars: AMF-treatment; Red bars: Nematode-treatment Grey bars: Control treatment (no belowground biota). Error bars represent the 99% CI for each of the selection line x treatment combinations. *ns: differences between nematode and AMF treatment are not significant after correction for multiple testing (pairwise difference: P = 0.01).

By integrating the above described variation in life history traits into one fitness measure (*r_m_*, here growth rate over one generation) significant differences according to the different selection line x soil treatment interactions are pronounced (Table 2; Fig. 3). Simulated growth rate was highest for mites from the AMF selection line developing on AMF plants and for mites from the control selection line reared on control plants (all t>11.1; P<0.001). Reciprocal effects for mites from the nematode selection line are only significantly different from the AMF treatment when taking into account within line comparisons (t = −2.56; pairwise P = 0.011), but not when corrected for all multiple comparisons (P = 0.207). Overall, growth rates differed according to the selection line (Table 2), with on average highest growth rates in the nematode selection line (3.41±0.01 se) compared to the reference (3.33±0.02se) and AMF line (3.13±0.01se). On average, mites performed worst when reared on plants subject to the nematode treatment (2.95±0.02se) relative to the AMF (3.28±0.01se) and reference treatment (3.52±0.02se).

## Discussion

Our results indicate that aboveground herbivores are able to adapt to plant phenotypes induced by a belowground biotic agent. The observed adaptation was driven by variable quantitative changes of the different separately studied life history traits (i.e. fecundity, longevity, sex-ratio, time to maturity). When using an integrate measure of fitness (i.e., growth rate), mite performance was highest on plants with the same belowground treatment as the one they experienced during selection. Only for the nematode treatment and selection line, the effect was tendentious when taking into account multiple comparisons. Strict treatment effects show that mite performance was lowest on plants with AMF and nematodes compared to plants grown on sterile soil.

Local adaptation was prominent when comparing performance on hosts with an AMF treatment and a control treatment without introduced biotic component, and tendentious when mites were selected on plants with belowground nematode herbivory. In our experiment, quantitative changes in plant nutritional quality, biomass and water content were observed. No detectable levels of cyanogenic potential were observed, so adaptation towards altered levels of chemical defence compounds is unlikely. After treatment with belowground biota, plant phenotypes changed in multiple, and non-correlated ways with respect to the measured structural and biochemical parameters. Moreover, the absence of cyanogenic potential does not rule out the prevalence of hitherto unidentified defensive metabolites. With that said, we are not able to assign one exact plant trait to be the driving force for the observed local adaptation. More likely, adaptive responses are due to multiple, mutually interacting changes in plant chemistry and structure [2], [25]. Although we have controlled for maternal effects by breeding mites from the different selection lines for two generations under identical conditions, it remains possible that the observed effects are under control of for instance epigenetic effects rather than driven by genomic changes under natural selection [58]. However, from an ecological point of view this does not alter the relevance of our findings, namely that below- and aboveground biota may interact with each other in an adaptive way.

Genetic trade-offs were found for time to maturity and survival, but relative differences between the different treatments are highly diverse. Fecundity was always higher on AMF plants compared to those on the nematode treatment. We only found genetic variation for sex-ratio and no evidence for any genetic trade-offs. However, a strong female biased sex ratio evolved in the mite population from the control selection line raised on control plants. Consequently, selection by host plants with different belowground treatments appears to be accompanied by variable quantitative changes in different life history traits. Instead, the integrated fitness measure *r_m_* is conclusive for the prevalence of local adaptation to belowground biotic conditions and the presence of genetic trade-offs [53].

Soil biota are documented to induce changes in population dynamics of their host and associated herbivores through changes in fitness [46]. These effects are either direct, affecting the quantity and quality of resources or indirect, through the release of carbon in the rhizosphere [19]. While mechanisms behind the interactions between foliar and root biota were explained in terms of water stress, primary chemistry and available biomass in early studies [22], [59] recent studies highlighted the importance of plant secondary metabolism as an explanation of both positive and negative feedbacks (reviewed in [23]). As demonstrated in our study, these belowground induced selection pressures may lead to local adaptation of the aboveground living herbivores to the host plants' specific belowground biotic conditions when exposure lasts over multiple generations. This finding is novel, and adds to the scarce literature on herbivore adaptation within single plant species. Leafminers, for instance were documented to be locally adapted to their host tree phenotype, despite often small distances between different plants under natural conditions [15], [16]. These tiny insects develop entirely within a leaf. As such, host phenotypic rather than genotypic heterogeneity due to variation in host-plant age and phenology are hypothesized to generate a coarse-grained spatially heterogeneous environment for the leafminer populations.

Belowground living species potentially show a similar strong spatial structure, although detailed knowledge on the scale and spatial structure is largely lacking in many natural systems [60]. As for abiotic soil conditions [61], the spatial contagion of the belowground biotic mosaic may therefore induce strong selection pressure on plants and their associated herbivores, with the potential for multiple-species coevolutionary dynamics [4]. Even in absence of coevolution between the hosts and the belowground biotic community, due to plant gene flow and dispersal, the latter may induce strong evolutionary specialization effects on spatially separated herbivores on the same host plant [62]. The belowground biotic community should consequently be acknowledged as a hitherto overlooked component for speciation of aboveground living herbivores. The strongest evolutionary changes were found between plants treated with AMF and those without any belowground treatment while less pronounced effects were found for mites raised on nematode inoculated plants. *Tetranychus urticae* was documented to adapt rapidly to host plant species with different nutritional and/or chemical constitution [46], [47]. Our experiment additionally demonstrates that more cryptic specialization towards changes in plant quality can equally well be induced by biotic conditions in the rhizosphere.

We here demonstrate that local adaptation of aboveground herbivores towards plant phenotypes influenced by belowground biota is possible. However, in nature many plant-associated species interact, both below- and aboveground [43]. So, probably only in rare situation this one-to-one situation may be significant under natural conditions and patterns of local adaptation towards plant phenotypes are expected to be determined by community-wide rather than single-species effects. This does, however, not alter our conclusions that aboveground herbivores may locally adapt towards plant phenotype with different belowground biota. Instead, average effects of the belowground community (in combination with effects mediated by their aboveground counterparts) are then expected to determine the plant phenotype. The only prerequisite for local adaptation to occur, is that biotic pressures on the plant population remain stable over time scales to allow evolutionary and co-evolutionary responses [4], [63]. This information is now largely lacking [60], [64], but we advocate that this kind of research is necessary to forecast evolutionary changes in plant-herbivore interactions at longer time frames, for instance within the framework of climate change or invasions.

In conclusion, we demonstrated that aboveground living arthropod herbivores are able to adapt to plant phenotypes induced by belowground biotic agents. These findings complement the few existing studies showing local adaptation of herbivores to specific geno- and phenotypes. However, we are the first to demonstrate evolutionary changes in populations of aboveground herbivores as a response to interactions with biota living on a spatially separated part of the same plant. This implicates that spatially homogeneous belowground communities can be expected to induce fast local adaptation of aboveground living herbivores, leading to increased growth. Because such conditions are expected to be met in current agricultural landscapes [65], fast local adaptation may consequently underlie pest dynamics of many typical crop herbivores. If true, restoration of belowground biotic heterogeneity can consequently be expected to slow down pest outbreaks.
